# An inducible offense: carnivore morph tadpoles induced by tadpole carnivory

**DOI:** 10.1002/ece3.1448

**Published:** 2015-03-04

**Authors:** Nicholas A Levis, Sofia de la Serna Buzón, David W Pfennig

**Affiliations:** Department of Biology, University of North CarolinaCB#3280, Chapel Hill, NC, 27599

**Keywords:** Induced morphology, inducible offenses, phenotypic plasticity, trophic polyphenism

## Abstract

Phenotypic plasticity is commonplace, and plasticity theory predicts that organisms should often evolve mechanisms to detect and respond to environmental cues that accurately predict future environmental conditions. Here, we test this prediction in tadpoles of spadefoot toads, *Spea multiplicata*. These tadpoles develop into either an omnivore ecomorph, which is a dietary generalist, or a carnivore ecomorph, which specializes on anostracan shrimp and other tadpoles. We investigated a novel proximate cue – ingestion of *Scaphiopus* tadpoles – and its propensity to produce carnivores by rearing tadpoles on different diets. We found that diets containing tadpoles from the genus *Scaphiopus* produced more carnivores than diets without *Scaphiopus* tadpoles. We discuss why *Scaphiopus* tadpoles are an excellent food source and why it is therefore advantageous for *S. multiplicata* tadpoles to produce an inducible offense that allows them to better utilize this resource. In general, such inducible offenses provide an excellent setting for investigating the proximate and evolutionary basis of phenotypic plasticity.

## Introduction

It is becoming increasingly apparent that an organism's environment can profoundly alter its phenotype (reviewed in West-Eberhard 2003; Gilbert and Epel 2009). Indeed, environmental cues, such as the food that an individual eats, can trigger completely different phenotypes within the same population. For example, in many Hymenoptera, when larvae are fed a rich diet (royal jelly), they develop into queens, but when they are fed a poor diet, they develop into morphologically distinct (and sterile) workers (Haydak 1970; Wheeler 1986). Understanding the proximate and evolutionary basis of such phenotypic plasticity is important, because these sorts of environmentally triggered alternative phenotypes represent some of nature's most dramatic examples of diversity within species (reviewed in Pfennig and Pfennig 2012). Moreover, environmentally initiated phenotypic change might precede, and even facilitate, genetic evolution (West-Eberhard 2003; Moczek et al. 2011; Laland et al. 2014).

A type of diet-induced plasticity that has been relatively understudied are “inducible offenses” (Padilla 2001; Kishida et al. 2009). Inducible offenses are defined as traits produced during an individual's lifetime in response to characteristics of a specific type of resource, which enhance the individual's acquisition of that (or similar) resource(s) (Padilla 2001; Kopp and Tollrian 2003; Mougi et al. 2011). For example, tiger salamanders (*Ambystoma tigrinum*) in the southwestern USA inhabiting temporary ponds without a top predator (i.e., fish) experience intense resource competition. Under the crowded conditions common within these ponds, some individuals facultatively develop a more robust head and larger teeth that facilitate cannibalism on relatively smaller, normal morphs (Collins and Cheek 1983; Collins and Holomuzki 1984; Pedersen 1991; Reilly et al. 1992; Hoffman and Pfennig 1999). Thus, the abundance of competitors (a characteristic of the resource) induces a morphological change in some individuals that allows them to prey on others (enhanced acquisition of the resource). Similarly, in response to its tadpole prey developing a “bulgy” body (a characteristic of the resource), the Ezo salamander (*Hynobius retardatus*) increases its gape size to facilitate ingestion of the larger tadpoles (enhanced acquisition of the resource) (Kishida et al. 2009). In this case, the predator-induced bulgy body induces a novel morphology in the predator. These examples conform to theoretical expectations, which hold that the particular external cues that trigger an environmentally induced phenotype should be correlated with the fitness of the induced phenotype (Levins 1968; Charnov and Bull 1977; Lively 1986). In the examples above, the induced offense takes advantage of, or overcomes some aspect of, the prey's phenotype.

Tadpoles of the spadefoot toad (*Spea multiplicata*) possess one of the best-studied inducible offenses. This species has two ecomorphs that utilize different food resources. The omnivore morph is the default phenotype and has adaptations that facilitate feeding primarily on organic detritus on the pond bottom: a long intestine, small jaw muscles, numerous labial teeth, and smooth mouthparts (Pomeroy 1981; Pfennig 1990). The carnivore morph, by contrast, feeds primarily on anostracan fairy shrimp and other tadpoles in the water column and has adaptations such as short intestines, greatly enlarged jaw muscles, few labial teeth, and notched mouthparts (Bragg 1965; Pomeroy 1981; Pfennig 1990, 1992). Each morph's distinctive features enable that morph to utilize its specific resource (Frankino and Pfennig 2001; Martin and Pfennig 2009).

The carnivore morph is environmentally triggered (Pomeroy 1981). Although numerous extrinsic and intrinsic factors contribute to the production of this morph (Pomeroy 1981; Pfennig 1992; Pfennig and Murphy 2000; Storz 2004; Pfennig and Martin 2009; Storz et al. 2011), theory predicts the most reliable trigger should be correlated with morph functionality. Consistent with this prediction, previous studies have shown that ingestion of fairy shrimp or conspecifics – common food sources of this morph – reliably predicts the carnivore phenotype (Pomeroy 1981; Pfennig 1990). Additionally, although large omnivores can eat shrimp, they are much less efficient than carnivores at doing so (Frankino and Pfennig 2001). Moreover, additional experiments have shown that the more extreme a carnivore is, the more effectively it can capture and consume live shrimp (Martin and Pfennig 2009). Thus, induction of the carnivore (offensive) phenotype corresponds to the functionality of the phenotype in acquiring the inducing resource.

**Figure 1:**
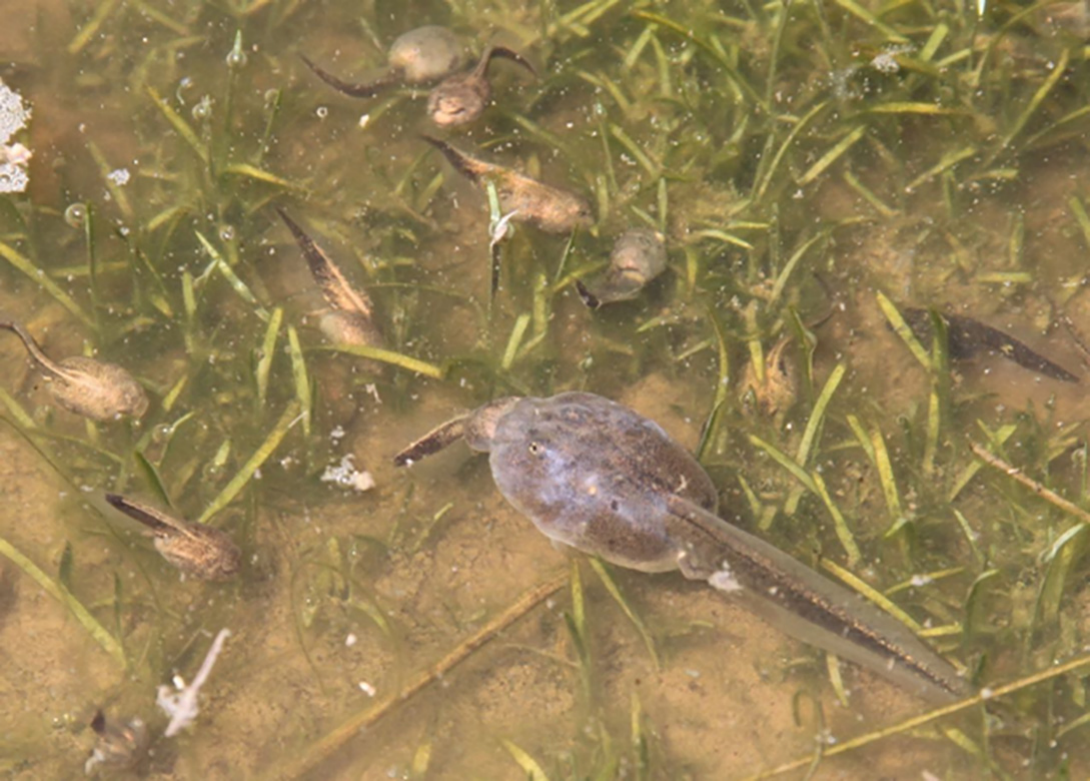
A carnivore morph *Spea multiplicata* tadpole eating a *Scaphiopus couchii* tadpole with additional *Sc. couchii* tadpoles nearby. Location of pond: Portal, AZ.

In the wild, however, carnivore tadpoles have another possible food source: heterospecific tadpoles. Indeed, controlled experiments revealed that *Spea* tadpoles grow and survive best on – and actually prefer to eat – tadpoles of the genus *Scaphiopus*, possibly because *Scaphiopus* tadpoles maximize nutrition for *Spea* tadpoles while simultaneously minimizing the risk of pathogen transmission (Pfennig 2000). Furthermore, *S. multiplicata* tadpoles have been observed eating *Scaphiopus* tadpoles in large quantities in the wild (e.g., see Fig.1; D. Pfennig, pers. obs.). Therefore, we hypothesized that ingestion of *Scaphiopus* tadpoles would induce the carnivore offense. To test this hypothesis, we reared *S. multiplicata* tadpoles on four different diets, including diets containing *Scaphiopus* tadpoles. Our results suggest that ingestion of *Scaphiopus* tadpoles is indeed a powerful cue for inducing the carnivore morph in *Spea* tadpoles.

## Methods

### Breedings and experimental design

We bred five pairs of *S. multiplicata* collected near Portal Arizona, USA, that had been part of an established laboratory colony at the University of North Carolina, Chapel Hill, for 1–2 years. These adults were from a high-elevation population, which does not co-occur with another species, *S. bombifrons,* and which therefore posses a relatively high propensity for producing carnivores (see Pfennig and Murphy 2002). Breeding was induced by injecting adults with 0.04 mL luteinizing hormone-releasing hormone (Sigma L-7134, St. Louis, MO, United States) at a concentration of 0.01 *μ*g/*μ*L and leaving pairs overnight in nursery tanks. Eggs from each sibship were kept in separate nursery tanks until tadpoles were free-swimming (Gosner stages 23-25), at which point individuals were divided haphazardly into one of four diet treatments (described below). Ten tadpoles were placed into rearing tanks (34 × 21 × 12 cm) filled with 3.5 L of dechlorinated tap water. Sixty rearing tanks per treatment were divided equally among three metal racks containing five shelves each. After 2 weeks, tadpoles were then evenly divided into smaller tanks (18 × 13 × 8.5 cm) containing 1.2 L of dechlorinated water to increase relative food availability to each tadpole (the same amount of food was given to each small tank as was given to the larger ones). Experimental units consisted of all tanks of the same treatment on the same shelf within a rack. Thus, the experiment consisted of three replicates of five families exposed to four treatments for a total of 60 experimental units where each experimental unit contained 40 individuals.

### Diet treatments

The experimental tadpoles were fed exclusively one of four different diets: (1) detritus; (2) live fairy shrimp; (3) live *Scaphiopus couchii* tadpoles; and (4) live fairy shrimp *plus* live *Scaphiopus couchii* tadpoles. When *Sc. couchii* tadpoles were depleted, we used similar-sized *Sc. holbrookii* tadpoles as prey for our experimental animals. Each tank in the detritus diet treatment was given ground fish food ad libitum. This resource is similar in composition to the tadpole's detritus food source in the wild (Pfennig et al. 1991).

Tadpoles in the shrimp diet treatment were fed live shrimp twice daily. Although *S. multiplicata* typically feed on Anostracan fairy shrimp in the wild, and these shrimp are known to induce the carnivore morph (Pfennig 1990), they are difficult to rear in large quantities in the laboratory. Therefore, we used a combination of fairy shrimp and brine shrimp (*Artemia* spp.), because brine shrimp are easily reared in large quantities and can potentially induce the carnivore phenotype in *Spea* (Ledon-Rettig et al. 2008). These tadpoles were fed 10-20 ml of concentrated fairy shrimp juveniles and nauplii each morning and 10–20 mL of highly concentrated brine shrimp juveniles and nauplii each evening. On those occasions when we were unable to feed fairy shrimp (due to a crash in our fairy shrimp colony), brine shrimp were used instead.

Tadpoles in the *Scaphiopus* diet treatment were given a 1:1 ratio of *Scaphiopus* tadpoles once daily. *Scaphiopus* tadpoles were held at higher densities and lower temperatures than the experimental *S. multiplicata* tadpoles to keep them at a smaller (more edible) size. In the majority of cases, *Scaphiopus* tadpoles were eaten almost immediately. Those that remained in the tanks for 24 h were replaced with smaller tadpoles that were then eaten. In tanks where *Scaphiopus* tadpoles often remained uneaten, few carnivores were produced, suggesting that tadpole consumption, rather than increased overall tadpole density, facilitated morph production. On 4 days, our stock of *Scaphiopus* tadpoles was depleted before a new stock of sufficient numbers to feed all the individuals was generated. On these days, tadpoles were fed the same diet as the shrimp treatment. Thus, the proportion of tadpole-fed to shrimp-fed days for this treatment was ∽5:1.

Tadpoles in the shrimp plus tadpole diet treatment alternated daily between the diet of the shrimp treatment and that of the tadpole treatment. The proportion of shrimp-fed to tadpole-fed days for this treatment was ∽1.5:1. After 9 days, the water was changed in all tanks and 20 mg of detritus was added as a supplemental food source.

For all treatments, multiple individuals were reared together in each tank, which created the possibility that some individuals monopolized food resources. This competition was expected – and potentially required (Pfennig and Frankino 1997) – for production of the carnivore morph. All procedures were performed in accordance with the University of North Carolina Institutional Animal Care and Use Committee Protocol Numbers 12-054.0 and 14.088.0.

### Response variables

After 20 days of feeding on the experimental diets, two of us independently scored each tadpole as an omnivore or a carnivore by qualitatively evaluating the size of each tadpole's jaw muscles relative to its body size, shape of its head, coloration, and foraging behavior (as carried out in other published work: Pfennig 1990, 1992; Pfennig and Frankino 1997; Pfennig 1999; Pfennig and Murphy 2000; Pfennig and Martin 2010). Morph assignments were unambiguous (i.e., only carnivores where the two scorers were in complete agreement were counted) and made without a priori knowledge of diet treatment (although the carnivores produced in this study were distinct from omnivores, they were generally less robust than wild-caught carnivores).

### Statistical analysis

The relationship among response variables and diet was evaluated using linear mixed-effects models fitted with restricted maximum likelihood in the lme4 package of R (Bates and Maechler 2009). Number of carnivores produced was fitted with a Poisson error distribution because it consisted of count data with many zeros. “Diet” was a fixed categorical variable and “family” a random effect. To ensure that diet treatments best explained the observed data, a full model containing both fixed and random effects was compared with a null model only containing an intercept and random effects using the “anova” function in the lme4 package in R.

After verifying that diet treatments best explained the variation in our data, we performed a nonparametric randomized residual permutation procedure (“RRPP”) to calculate effect sizes between groups and to identify between-group differences (Collyer and Adams 2007; Collyer et al. 2014). This procedure extracts the residuals of a null model and randomly pairs them with fitted values; then, these pseudorandom data are used to calculate pairwise distances using the full model. By repeating this process 5000 times, we were able to determine the probability of finding differences greater than or equal to the observed distances (D_obs_) between group means. Essentially, this procedure acts like an ANOVA with a multiple comparisons test, but is not constrained by the assumptions associated with a parametric procedure. Similar to an ANOVA, this procedure generates an F statistic that is the ratio of error variance between the reduced and a full model and the error variance of the full model, which quantifies the variation explained by the addition of the treatment parameter, diet, in the full model. All analyses were performed using R version 3.1.2 (R Core Development team; http://www.r-project.org/) with *α *= 0.05.

## Results

**Figure 2:**
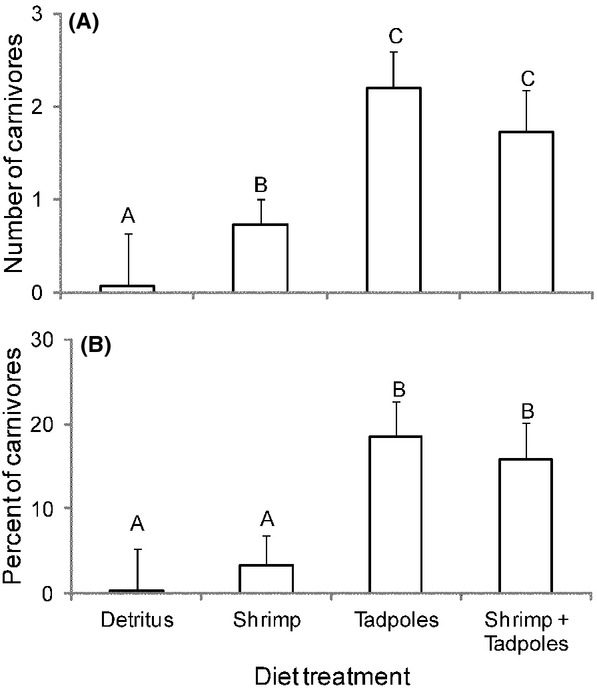
Mean (+SEM) number of *S. multiplicata* tadpoles that developed into carnivores (A) and proportion of survivors that became carnivores (B) in each diet treatment. Different letters denote significant differences among groups (*P* < 0.05).

In all cases, the full model containing diet as a fixed effect and family as a random effect was significantly better than a null model only containing an intercept and the random effect (Table1). As with previous studies, the shrimp-only diet produced more carnivores than the detritus treatment. In addition, tadpole and shrimp plus tadpole diet treatments produced a significantly higher number and proportion of carnivores compared to shrimp only and detritus diet treatments (Table2; Fig.2).

**Table 1:** Model comparison and summary statistics for number and proportion of carnivore morph tadpoles produced using AICc values and log likelihood. “Number of carnivore” models were fit to a Poisson distribution. Best-fit models are bolded. All models contained clutch as a random effect.

	df	ΔAICc	log LiK	*χ*^2^	*R*^2^	*F*	*P*
Number of Carnivores							
Null	2	30.18	−89.746				
**Diet**	**5**	**0**	−**67.497**	**44.50**	**0.54**	**21.55**	**1.183E-09**
Proportion of Carnivores							
Null	3	1.9	22.225				
**Diet**	**6**	**0**	**30.36**	**16.27**	**0.26**	**6.53**	**0.001**

df indicates degrees of freedom, ΔAICc is the change from lowest AICc value, log Lik is log likelihood.

**Table 2:** Observed distances (A) and p-values (B) for pairwise distances among treatments for number (top) and proportion (bottom) of carnivores produced based on a nonparametric randomized residual permutation procedure (“RRPP”) with 5000 iterations. Bold values are significant with *α *= 0.05.

	Detritus	Shrimp	Tadpoles
Number of carnivores produced			
(A) D_obs_			
Shrimp	**2.40**	–	–
Tadpoles	**3.50**	**1.10**	–
Shrimp + Tadpoles	**3.26**	**0.86**	0.24
(B) *P*-values			
Shrimp	**0.0002**	–	–
Tadpoles	**0.0002**	**0.0094**	–
Shrimp + Tadpoles	**0.0002**	**0.0368**	0.5830
Proportion of carnivores produced			
(A) D_obs_			
Shrimp	0.03	–	–
Tadpoles	**0.18**	**0.15**	–
Shrimp + Tadpoles	**0.16**	**0.13**	0.03
(B) *P*-values			
Shrimp	0.6238	–	–
Tadpoles	**0.0004**	**0.0044**	–
Shrimp + Tadpoles	**0.0036**	**0.0202**	0.6706

## Discussion

As predicted, diets containing heterospecific tadpoles induced the carnivore phenotype more frequently than those that did not. Because *S. multiplicata* tadpoles grow and survive best on a diet of *Scaphiopus* tadpoles (Pfennig 2000), it makes sense that ingestion of this resource would induce phenotypic changes that allow for presumably greater utilization of the resource (i.e., this is an induced offense). Indeed, the distinctive carnivore morph is specialized for pursuing and subduing large, mobile prey, such as *Scaphiopus* tadpoles (Martin and Pfennig 2009).

Previous work established that the ingestion of live fairy shrimp (Anostraca) induces the carnivore phenotype as well (e.g., Pomeroy 1981; Pfennig 1990, 1992). Consistent with this previous research, we observed a significant difference in the average number of carnivores produced between the detritus and shrimp diets (1 vs. 11; Fig.2). In accord with our expectations, tadpole-fed animals produced more carnivores than either the detritus or shrimp-only groups. Although our shrimp diet (a mixture of brine and fairy shrimp) might not have been as effective at triggering carnivores as a natural diet of pure fairy shrimp, our data indicate that *Scaphiopus* consumption is the more effective cue at inducing carnivores.

We found no evidence to indicate that either the total amount or the nutritional quality of a particular diet contributed to carnivore production. Of the four diet treatments, the detritus-fed tadpoles received the greatest biomass of food, but these tadpoles produced the fewest number of carnivores. Furthermore, tadpoles reared on the shrimp diet may have had access to a greater biomass than tadpoles reared on diets containing other tadpoles. However, tadpoles reared on other tadpoles produced the most carnivores. It is possible that the quality of a single tadpole is greater than that of several shrimp. Yet, Pfennig (1992) noted that tadpoles fed a diet of 46% protein did not produce more carnivores than tadpoles fed a diet of only 6% protein. Thus, it appears that some factor other than biomass or protein content is responsible for carnivore induction.

*Scaphiopus* tadpoles are an excellent food source for *S. multiplicata* tadpoles, for at least five reasons. First, as noted above, *Scaphiopus* tadpoles might optimize a possible trade-off between nutrition and pathogen acquisition (Pfennig 2000). Specifically because they are of intermediate phylogenetic similarity to *S. multiplicata* (compared to conspecifics on the one hand and shrimp on the other hand), *Scaphiopus* tadpoles are compositionally similar in proportions of materials necessary for growth, maintenance, and reproduction, yet immunologically distinct enough to reduce the risk of transmitting contagions. Presumably, this explains why, compared to diets of detritus, shrimp, or conspecifics, *Spea* tadpoles grow and survive best on, and also prefer to eat, *Scaphiopus* tadpoles (Pfennig 2000). Second, the average clutch size of *Scaphiopus couchii* in the southwestern United States is approximately three times that of *S. multiplicata* (Woodward 1987), suggesting that *Scaphiopus* tadpoles are an abundant potential food source. Third, *Scaphiopus* tadpoles tend to be smaller than *S*. *multiplicata* tadpoles (Strecker 1908; Newman 1987; Pfennig et al. 1991; Degenhardt et al. 1996), which should make them relatively easy to subdue and consume (see also Fig.1). Fourth, *Scaphiopus* larvae form aggregations (Black 1973), which reduces search time and increases efficiency of predation by *S. multiplicata* (see Fig.1). Therefore, several characteristics of *Scaphiopus* tadpoles make them an excellent food source for *S. multiplicata* tadpoles.

The present study has revealed several avenues for further investigation. First, verification that carnivores are better at handling and consuming *Scaphiopus* tadpoles than omnivores is needed. We assumed that because carnivores are better at handling and consuming fairy shrimp and conspecifics (Pomeroy 1981; Pfennig and Murphy 2000) – which are smaller and larger, respectively, than *Scaphiopus* tadpoles – they should be better at consuming *Scaphiopus* tadpoles as well. Yet, this assumption needs to be tested.

Additionally, further studies are needed to determine whether different diets influence the degree to which tadpoles express the carnivore morphology. In the present study, we were simply interested in evaluating the possible role of different diets on inducing *any* carnivores. Yet, some diets might produce more extreme carnivores than others (e.g., higher concentrations of shrimp induce more extreme carnivores than lower concentrations; see Pfennig 1990). We might expect that ingesting tadpoles would generate not only more carnivores but also more morphologically extreme carnivores, because the ingestion of a tadpole would likely require larger jaw musculature and mouthparts than the ingestion of the smaller shrimp.

Finally, future studies should compare carnivore production between tadpoles fed phylogenetically close tadpole species versus those fed phylogenetically distant tadpole species. Generally, phylogenetically close species might represent a greater competitive threat than phylogenetically distant tadpole species (e.g., see Violle et al. 2011). Moreover, they might be more nutritious (e.g., see Pfennig 2000). Thus, compared to phylogenetically distant species, phylogenetically close species might tend to more frequently trigger inducible offenses (such as carnivores) as well as generate more extreme versions of those offenses.

In sum, the carnivore phenotype of *Spea* tadpoles fits the definition of an inducible offense in that it is environmentally triggered by diet and allows individuals to take further advantage of an abundant, nutritious resource. Understanding the proximate and evolutionary basis of diet-induced plasticity is important, because such plasticity might play an underappreciated role in the origins of diversity.

## Data Accessibility

Data is available as online supporting information (Table S1).

## Supporting Information

**Table S1**. Raw data that accompanies this manuscript.
